# Bioactive Polymeric Materials for Tissue Repair

**DOI:** 10.3390/jfb8010004

**Published:** 2017-01-26

**Authors:** Diane R. Bienek, Wojtek Tutak, Drago Skrtic

**Affiliations:** 1 Volpe Research Center, ADA Foundation, Gaithersburg, MD 20899, USA; wojtek.tutak@fda.hhs.gov (W.T.); drago.skrtic@nist.gov (D.S.); 2 Food and Drug Administration, Silver Spring, MD 20993, USA

**Keywords:** airbrushing, blow spinning, amorphous calcium phosphate, bone repair, cell/fiber interactions, nanofibers, remineralizing polymeric composite

## Abstract

Bioactive polymeric materials based on calcium phosphates have tremendous appeal for hard tissue repair because of their well-documented biocompatibility. Amorphous calcium phosphate (ACP)-based ones additionally protect against unwanted demineralization and actively support regeneration of hard tissue minerals. Our group has been investigating the structure/composition/property relationships of ACP polymeric composites for the last two decades. Here, we present ACP’s dispersion in a polymer matrix and the fine-tuning of the resin affects the physicochemical, mechanical, and biological properties of ACP polymeric composites. These studies illustrate how the filler/resin interface and monomer/polymer molecular structure affect the material’s critical properties, such as ion release and mechanical strength. We also present evidence of the remineralization efficacy of ACP composites when exposed to accelerated acidic challenges representative of oral environment conditions. The utility of ACP has recently been extended to include airbrushing as a platform technology for fabrication of nanofiber scaffolds. These studies, focused on assessing the feasibility of incorporating ACP into various polymer fibers, also included the release kinetics of bioactive calcium and phosphate ions from nanofibers and evaluate the biorelevance of the polymeric ACP fiber networks. We also discuss the potential for future integration of the existing ACP scaffolds into therapeutic delivery systems used in the precision medicine field.

## 1. Introduction

The field of biomedical material research—the ultimate goals of which are to repair, preserve and/or advance functions of tissues damaged by pathological conditions and/or trauma—has advanced profusely in the last several decades. Presently, potential exists for regenerating almost every human tissue and/or organ [1]. It is generally accepted that biomaterials, by either providing adequate bio-environment or just a support, play a pivotal role in tissue regeneration. In terms of their biological, compositional, mechanical, and structural properties, biomaterials can be categorized as bio-inert or bioactive ceramics, glasses, and polymers.

Calcium phosphates (CaP) are of significant interest to the biomedical and dental fields due to their involvement in both normal, as well as the pathological, mineralization (urinary and dental calculus) and demineralization (dental caries) [2,3,4]. Bioactive, CaP-based materials [3] and bio-glasses [4,5], via reaction with physiological fluids and/or through cellular activity, bond firmly to both hard and soft tissues. Among CaPs, amorphous calcium phosphate (ACP) is a unique non-crystalline compound which, due to its thermodynamic instability in aqueous environments, spontaneously transforms into crystalline orthophosphates, mainly hydroxyapatite (HAP) [6]. ACP forms instantaneously during the spontaneous precipitation from supersaturated basic calcium (Ca) and phosphate (PO_4_) solutions. Its structural, chemical, and thermodynamic properties have been extensively discussed in review articles [7,8]. HAP is a thermodynamically stable form of CaP in neutral and basic environments. The rate of ACP conversion to HAP depends primarily on the chemistry of the microenvironment. As a result of this conversion, the crystallinity of the solid and its Ca/PO_4_ ratio increase with time. The process can be affected by the presence of inorganic anions, cations, or organic molecules which can adsorb on the ACP surface, incorporate into the ACP structure and/or co-precipitate with ACP.

Once exposed to oral fluids, ACP-filled composites release Ca and PO_4_ ions (natural building blocks of tooth minerals) and create supersaturation conditions for the regeneration of tooth structures lost to decay and/or wear. ACP’s documented role in matrix vesicle mineralization, and its chemical similarity to the mammalian calcified tissues, accentuates its role as a key intermediate in skeletal calcification and make ACP a promising candidate to manufacture bone grafts [7].

Both the osteoconductivity and biocompatibility of CaP-based biomaterials designed for dental and/or orthopedic bone tissue regeneration vary with the type of CaP utilized. CaPs with solubility above that of HAP are reactive and expected to contribute to bone formation with the aid of osteoblasts. However, despite considerable research efforts, the exact mechanism by which these more soluble CaPs promote osteogenesis remains unclear [9,10,11,12]. The majority of CaP cements are formulated from the solutions containing no ions other than Ca, PO_4_, Na, and/or K. Ca-regulated cell responses and the osteoblast-mediated transport of PO_4_ are recognized as the prerequisites for bone mineralization [13,14,15] and cellular receptors for both Ca and PO_4_ have been identified [14,16]. It has also been documented that a transient, amorphous precursor may be essential for mineral formation in the supersaturated in vivo environments [17,18]. Furthermore, ACP has been suggested as a required intermediate phase in HAP nanocrystal formation [19,20] and identified in newly-formed enamel [21]. These discoveries have helped us to better understand the role of ACP in in vivo biomineralization and stimulated our group to include the ACP in design of novel materials intended for tooth [22] and bone tissue preservation and/or regeneration [23].

Natural polymers, such as proteins, polysaccharides, or polynucleotides [24], typically interact favorably with cells. Synthetic polymers, on the other hand, offer a possibility of tailoring their physicochemical and mechanical properties for specific applications. Two main areas of their biomedical utility are (1) carriers in drug delivery applications; and (2) promoters of cell growth and organ healing in cell scaffolds and other tissue engineered materials. Among multiple concerns related to the cell-guided tissue regeneration, the effect of morphology of nanofiber scaffolds on cellular responses has been highlighted and the importance of cell-material compatibility, promotion of cell growth, inter-cell communication and 3D architectural characteristics of the constructs emphasized [25,26,27].

### 1.1. Objectives

An objective of this study is to present the development and characterization of the bioactive, ACP-based remineralizing composites for dental applications. We consider how ACP’s dispersion in polymer matrix and fine-tuning of the resin affect the physicochemical, mechanical, and biological properties of ACP polymeric composites.

For proper hard tissue healing, it has been well documented that bioavailable Ca and PO_4_ are essential [28]. Convincing evidence has also been presented indicating ACP as supportive of cell attachment, growth, and differentiation [29,30,31,32]. Our group has developed an alternative to traditional electrospinning by advancing airbrushing (synonymous with blow spinning) as a platform technology that is capable of synthesizing open structure nanofibers at high rates [33,34,35]. As a second objective, we focused on (1) feasibility of incorporating ACP into various polymer fibers and assessing the release of Ca and P from these composite materials; and (2) evaluation of cellular responses to these materials. We also discuss the potential for ACP utility in the precision medicine field.

## 2. Results

### 2.1. Polymeric Bioactive ACP Dental Composites

For over two decades, our group has been developing polymeric dental composites that utilize ACP as a bioactive filler [32,36,37,38]. Due to the intrinsically low strength and modulus and, thus, poor reinforcing ability, the utility of ACP composites is limited to non-load-bearing applications. However, ACP’s unique ability to counteract recurrent decay (up to 50% of all dental fillings require replacement because of recurrent caries) makes these ACP composites still highly attractive for use in cavity-prone patients who experience oral dryness due to salivary gland malfunctioning, radiation therapy, and/or use of certain medications. The underlying working hypothesis in our structure/composition/property studies is that by surface modification of the filler phase in conjunction with the fine tuning of the resin phase, a desirable physicochemical, mechanical, and biological performance of ACP composites is achievable.

#### 2.1.1. Physicochemical and Mechanical Properties

**Figure 1:**
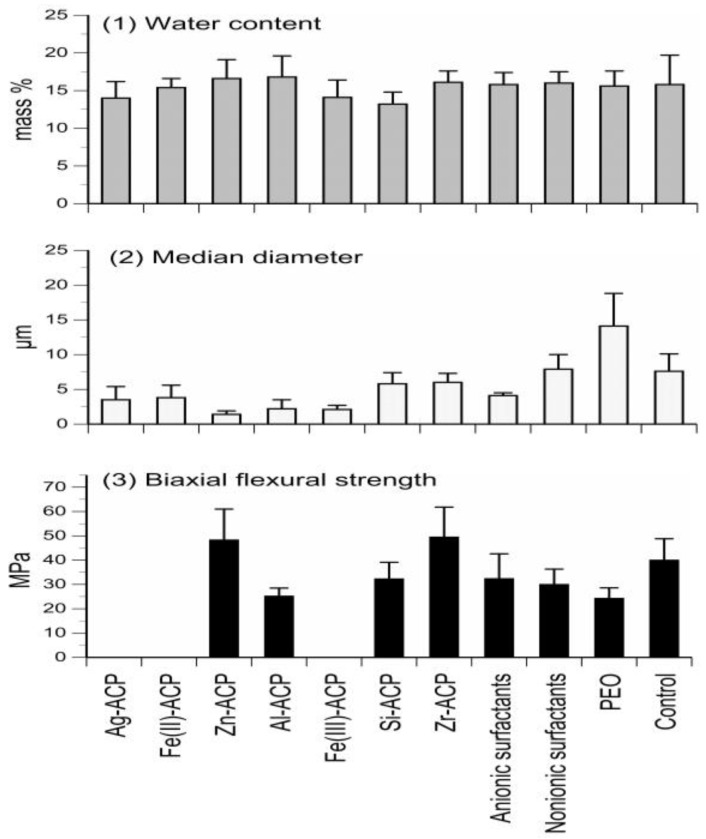
Effects of additives (cations, surfactants, and polymers) on ACP filler’s water content (**upper panel**), particle size (**middle panel**), and mechanical strength (**lower panel**) of the Bis-GMA-based composites. Indicated are mean values + standard deviation (SD) for three or more repetitive runs in each experimental group.

To yield ACP solids with homogeneous particle size distribution and, in turn, reduce the unfavorable uneven distribution of the filler within ACP composites, various additives (cations, surfactants and/or polymers) were introduced during ACP syntheses. The results of the studies focusing on filler modification are compiled in Figure 1.

The average overall water content (15.7 mass %) and the average ratio of surface-bound vs. the structurally incorporated water (2.33) was unaffected by the type of additive utilized (Figure 1; upper panel). X-ray diffraction analyses of the powders indicated that with silver-modified ACP (Ag-ACP) and the Fe-ACPs series, a light-sensitive silver-phosphate and colored Fe-phosphates co-precipitated with ACP, respectively. Additionally, the Fe-ACPs prematurely converted to HAP (data not shown). The reduced median particle size diameter and the accompanying narrower range of particle size distributions seen in zinc-modified ACP (Zn-ACP) and aluminum-modified ACP group appear random rather than related to the change of cation’s ionic potential. In the surfactant group, a 45% reduction in median particle size diameter compared to no-additive control was seen only with the anionic fluoro-surfactant. Addition of polymer, polyethylene oxide, however, had an inverse effect on ACP particle size resulting in 90% increase in median particle size diameter (Figure 1; middle panel). With the exception of Zn-ACP and zirconia-modified ACP (Zr-ACP), the biaxial flexure strength (BFS) of all cation-modified ACP 2,2-bis[p-(2-hydroxy-3-methacryloxypropoxy)phenyl]propane (Bis-GMA)-based composites was inferior to control ACP (Figure 1; lower panel). More specifically, the reduction in BFS ranging from 20% to 40% compared to control ACP was seen in all additive groups, with the exception of Zn-ACP and Zr-ACP. Composites formulated with Ag-ACP and Fe-ACPs completely disintegrated upon immersion. Since Zr-ACP based composites also showed significant (*p* < 0.000001) improvements in degree of vinyl conversion (DVC) and remineralization capacity (data not shown), Zr-ACP was chosen as “gold standard” filler and was used in all subsequent studies reported in this article.

**Figure 2:**
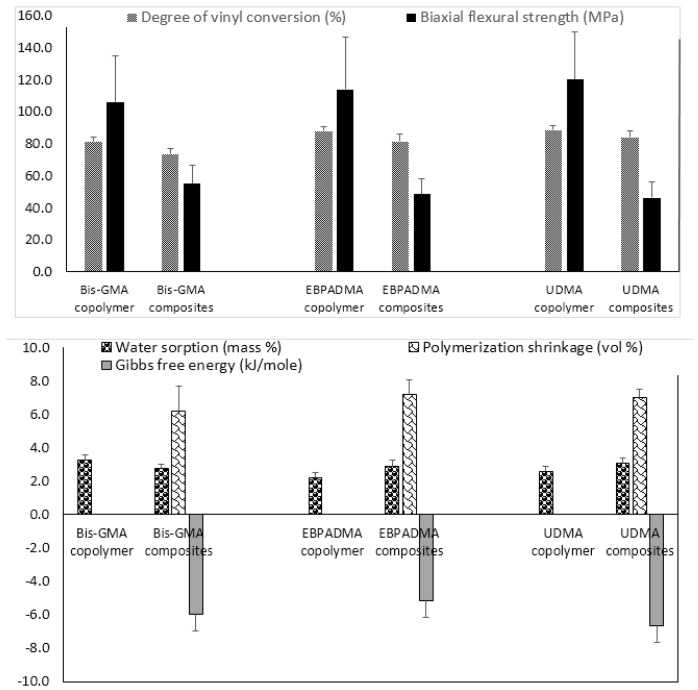
Physicochemical properties of Bis-GMA-, EBPADMA-, and UDMA-based experimental resins and their Zr-ACP composites intended for base/lining, orthodontic and endodontic utility, respectively. Indicated are mean values + SD. The number of repetitive runs/experimental groups are as follows: 24 ≤ *n* ≤ 60 (DVC), 16 ≤ *n* ≤ 43 (BFS) (**upper panel**); 12 ≤ *n* ≤ 39 (WS), 14 ≤ *n* ≤ 42 (PS), 4 ≤ *n* ≤ 16 (ΔG°) (**lower panel**).

Fine-tuning of the resin by adjusting polymer’s hydrophilicity/hydrophobicity to ensure a desirable ion release, changing ratio(s) of base, diluent, and surface active monomers to control matrix’s viscosity and enhance adhesiveness of the restoration to tooth structures, and selecting polymerization initiators to attain satisfactory levels of DVC, is another approach used in design of ACP composites. Implementation of this approach requires an extensive evaluation of both unfilled resins (copolymers) and the corresponding ACP-filled composites. The results of the evaluation of the experimental remineralizing composites formulated with Bis-GMA, ethoxylated bisphenol A dimethacrylate (EBPADMA) or urethane dimethacrylate (UDMA) as base monomers, triethylene glycol dimethacrylate (TEGDMA), 2-hydroxyethyl methacrylate (HEMA), hexamethylene dimethacrylate (HmDMA), and/or poly(ethylene glycol) extended-UDMA (PEG-U) as diluent monomers, and zirconyl dimethacrylate and/or methacryloyloxyethyl phthalate (MEP) as surface active, adhesion-promoting components are presented in Figure 2.

Generally, all experimental copolymers and composites attained relatively high levels of DVC 24 h post-curing. The values were 81.4%–88.6% and 73.5%–83.9% in copolymer and composite series, respectively (Figure 2; upper panel). Introduction of ACP filler into resins resulted in modest, though highly significant (two-sample independent *t*-test), reduction of DVC in all systems: 9.7% in the Bis-GMA group (*p* < 0.0000001), 7.1% in the EBPADMA group (*p* < 0.000003), and 5.4% in the UDMA group (*p* < 0.00007). Inter-group comparisons revealed a consistently lower DVC values in Bis-GMA- vs. EBPADMA- and/or UDMA-based formulations in copolymer series (7.3%–8.2% reduction; one-way analysis of variance (ANOVA): *p* < 0.0001) and (9.0%–12.4% reduction; one-way ANOVA: *p* < 0.0001) in composite series. The BFS of copolymer specimens upon aqueous immersion showed no dependence on resin composition. The apparent increase in BFS in going from Bis-GMA- to EBPADMA-, to UDMA-based copolymers was not statistically significant (one-way ANOVA: *p* > 0.05). Composite vs. copolymer comparison within the groups, indicated substantially lower BFS in composite series. The reduction in BFS in going from copolymer to composite ranged from 48% (Bis-GMA formulations), to 58% (EBPADMA formulations), to 61% (UDMA formulations). Data significance was confirmed by two-sample independent test (*p* values < 0.00004 for all comparisons).

With the attained DVC exceeding 73% in all composite systems, the polymerization shrinkage (PS) in these materials was, expectedly, relatively high, i.e., (6.2–7.2) vol % and practically independent of the resin composition (Figure 2; lower panel). Additionally, practically independent of the resin composition was the overall WS: maximum WS values ranged from 2.2 to 3.3 mass % for copolymers and 2.6% to 3.1% for composites. In all groups, the relative portions of hydrophilic (TEGDMA or HEMA; WS increased) or hydrophobic (HmDMA; WS decreased) affected the water uptake by copolymers and composites. Immersed in aqueous medium, all experimental composites yielded solution conditions conducive to HAP precipitation: calculated Gibbs free energy (ΔG°) values from -5.2 to -6.7 kJ/mol were highly above the minimum necessary for HAP formation i.e., ΔG° < 0.

#### 2.1.2. Extraction of Unreacted Monomers

**Figure 3:**
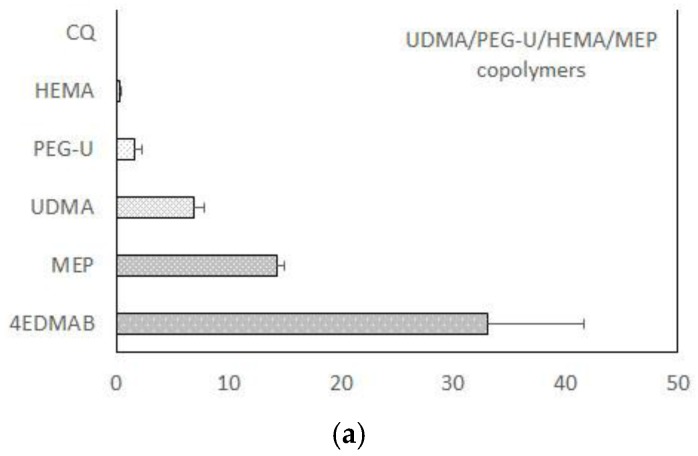

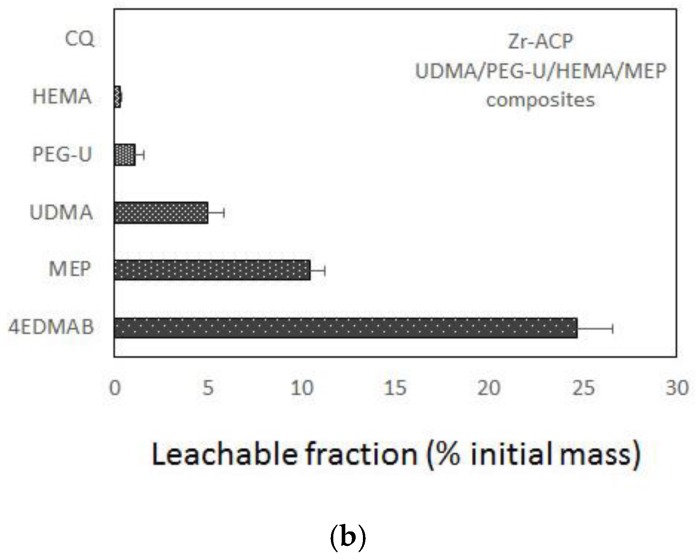
Leachability (acetone extraction; ^1^H NMR spectroscopy data) of unreacted monomers and constituents of the light-cure initiating system from UDMA-based copolymers (**a**) and Zr-ACP-based composites (**b**) formulated for endodontic utility. Indicated values represent mean values + SD for *n* = 3/experimental group.

Results of the accelerated leachability study (extractant: acetone) of the experimental endodontic sealant formulated from UDMA/PEG-U/HEMA/MEP (UPHM) resin and Zr-ACP are presented in Figure 3. The amounts of the leachable monomers (expressed as the percent of that was initially incorporated in the resin) detected by nuclear magnetic resonance (^1^H NMR) spectroscopy in both copolymer and composite series increased in the following order: HEMA < PEG-U < UDMA < MEP. Only marginal differences seen between the copolymer and composite leachability profiles suggest that elution of the unreacted species from this highly cross-linked resin was unaffected by introducing the remineralizing ACP filler to the resin phase.

#### 2.1.3. Remineralization Efficacy

**Figure 4:**
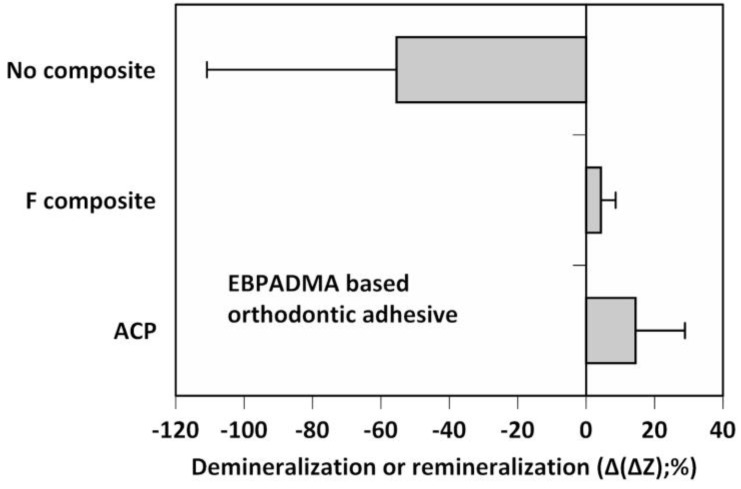
Microradiographic quantification of the overall mineral loss (negative Δ(ΔZ) values) or mineral recovery (positive Δ(ΔZ) values) in human teeth treated with experimental EBPADMA-based ACP orthodontic adhesive, commercial fluoride-releasing composite (F composite), or with no composite. Shown are mean Δ(ΔZ) values for a minimum of 32 microradiographic areas/group. SD values varied from 13.92% to 26.08%.

Remineralization efficacy of the experimental ACP composite formulated for orthodontic adhesive applications was assessed in a quantitative microradiographic study, which employed demineralized human enamel specimens that underwent pH cycling regimens to mimic acid challenges in oral milieu (Figure 4).

EBPADMA-based composites exhibited greater remineralization activity compared to controls. The overall remineralizing effect of ACP EBPADMA composite was three-fold greater than the remineralizing effect of the fluoride control (mean Δ(ΔZ) values of 14.44% vs. 4.31%, respectively) (Figure 4). However, due to a large data scattering in both experimental groups, this apparent change was found statistically insignificant (*p* = 0.107). Compared to no-composite controls that exhibited further mineral loss (mean Δ(ΔZ) = −55.44%), remineralizing efficacy of ACP EBPADMA composites was significantly higher (*p* < 0.05).

### 2.2. Airbrushed, ACP Nanofiber Scaffolds

#### 2.2.1. ACP Incorporation and Ion Release from Scaffolds

**Figure 5:**
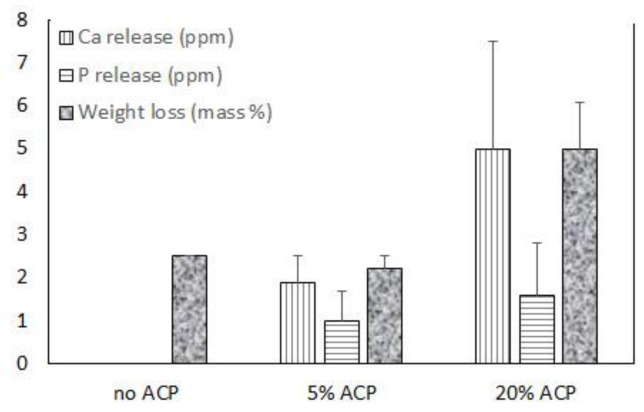
Ion release and a total weight loss in ACP PDLLA nanofibers and no-ACP control after four weeks of saline immersion. Indicated are mean values + SD (*n* = 4).

**Figure 6:**
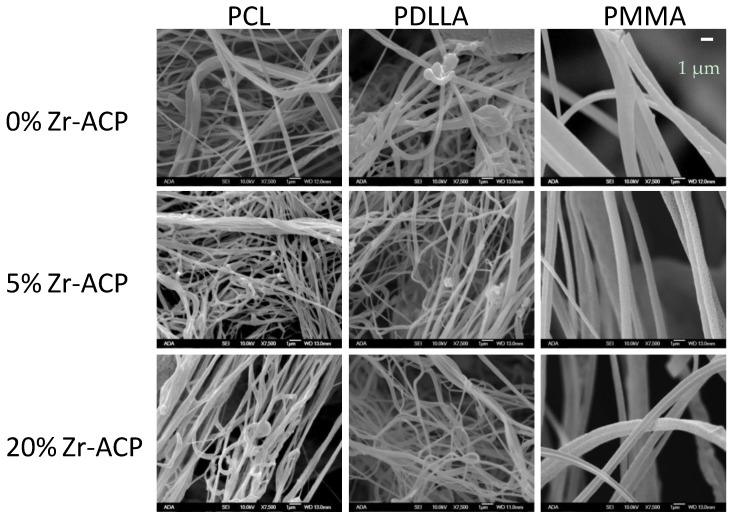
Morphology of airbrushed polymeric nanofiber scaffolds under scanning electron microscope (×7500 magnification). Scaffolds were fabricated using PCL, PDLLA, or PMMA blended with 0%, 5%, or 20% Zr-ACP.

Up to 20 mass % Zr-ACP was successfully incorporated in polymeric nanofibers (Figure 5 and Figure 6). Ion release from the scaffold was accessed as an indicator of the remineralization potential. This aspect was explored in poly-d,l-lactic acid (PDLLA) nanofiber scaffolds containing 0%, 5%, or 20% Zr-ACP. Control composite scaffolds without incorporated ACP exhibited low (2.5%) total weight loss after four weeks of immersion in saline solution similar to 5% ACP PDLLA series. In contrast, 20% ACP PDLLA fibers lost significantly (one-way ANOVA: *p* < 0.005) more (5% of the initial mass) upon immersion (Figure 5). This relative high weight loss in 20% ACP series, accompanied by the highest Ca and P release, is most likely caused by the more extensive structural decomposition compared to the fibers containing 5% ACP or no-ACP control. This conclusion is further supported by the optical micrographs of the immersed fibers which consistently indicated greater morphological changes in fibers with 20% ACP compared to the ones with 5% or no ACP (data not shown).

#### 2.2.2. Cellular Responses to ACP Polymeric Nanofibers

**Figure 7:**
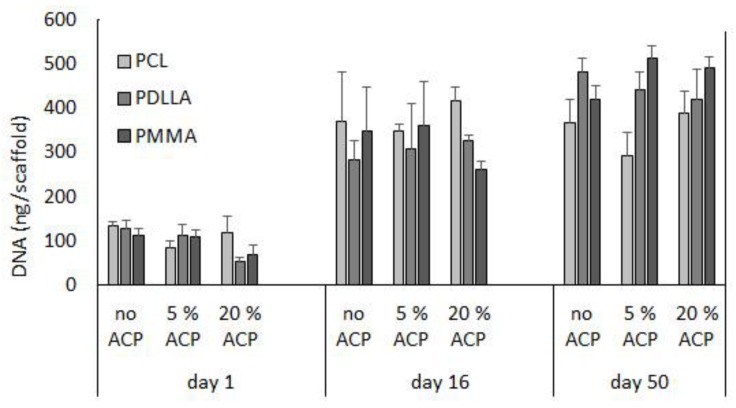
Effect of time (days 1, 16, and 50) and type of polymer (PCL vs. PDLLA vs. PMMA) on cell DNA content in ACP scaffolds vs. no ACP controls. Bar height indicates mean values + SD (*n* = 3).

To assess the biorelevance of the polymer fiber network, human bone marrow stromal cells (hBMCSs) were seeded on the polymeric nanofiber scaffolds. Hoffman et al. [23] reported that long-term cultivation of hBMSC on airbrushed composite scaffolds with different Zr-ACP loads (up to 20% (*w*/*w*)) largely demonstrated similar DNA content patterns. Further analyses, in the present study, indicate that, within a given Zr-ACP concentration, cellular DNA levels can vary with polymer type (Figure 7). For example, comparing polymers within given day and ACP group led to the following conclusions: in 20% ACP group, DNA levels decreased at day 1 in going from polycaprolactone (PCL) to PDLLA (*p* = 0.04) and at day 16 in going from PCL to PDLLA to poly(methyl methacrylate) (PMMA) (*p* ≤ 0.03). The trend was reversed in no-ACP and 5% ACP group at day 50, where a significant increase in DNA content was seen in going from PCL to PDLLA (*p* = 0.03) and from PCL to PDLLA to PMMA (*p* ≤ 0.01), respectively. Further, DNA content (inferring cell viability) increased with time. Within a given polymer and ACP level, DNA content increased in the following order: day 0 < day 16 ≤ day 50 (*p* ≤ 0.03).

**Figure 8:**
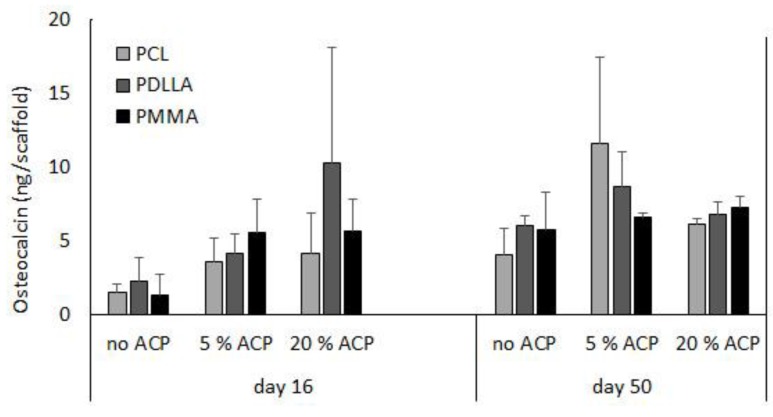
Effect of incubation time (days 16 and 50) and type of polymer (PCL vs. PDLLA vs. PMMA) on osteocalcin expression in ACP scaffolds vs. no ACP controls. Bar height indicates mean values + SD (*n* = 3).

A previous report indicated that long-term cultivation of hBMSC on airbrushed composite scaffolds with different Zr-ACP loads (0%, 5%, or 20% (*w*/*w*)) largely demonstrated osteocalcin protein levels that were not statistically different within a given day and polymer type [23]. Further analyses indicated that within a given day, the osteocalcin protein levels were not affected by the type of polymer carrier (*p* ≥ 0.09) (Figure 8). Moreover, comparison of osteocalcin expression between day 16 and day 50 revealed statistically significant increase in no ACP PDLLA group (*p* ≤ 0.02) and a marginal increase in the 5% ACP PDLLA group (*p* ≤ 0.05).

## 3. Discussion

### 3.1. Polymeric Bioactive ACP Dental Composites

Due to highly heterogeneous particle size distribution (uncontrolled aggregation) of ACP filler, ACP-based composites often exhibit poor filler/resin interface [38]. To improve the performance of ACP composites in an oral environment, it is important to achieve an even distribution of ACP particles within the polymer phase. In addition to improving the mechanical performance of the composites, more homogeneous filler/matrix interface will yield better control of the WS kinetics and the required release of remineralizing ions and the unwanted leaching of degradation products. ACP surface modification (Figure 1) generally did not result in a significant fillers’ homogenization and particle size reduction sufficient to improve composite’s mechanical properties. In cation series, the random reduction in particles’ median particle diameter could not be correlated with the ionic potential of the cations, i.e., ionic radius, multiplicity of charge, and/or water coordination. Introduction of surfactants and polymers had practically an inverse effect on particle size distribution and their addition compromised mechanical stability of the composites. Therefore, the alternative physical methods, i.e., grinding and milling of ACP filler, were explored and yielded improved BFS of composites upon aqueous immersion (data not shown). An enhanced hydration that likely caused the accelerated water diffusion into as-made ACP composites is apparently repressed in ground ACP and milled ACP systems. Significantly, even with the reduced WS, ground ACP, and milled ACP composites maintained satisfactory ion release, thus preserving the composites’ remineralization potential.

The conventional dental composites typically entail two distinctive phases, i.e., polymer matrix (predominantly methacrylate-based) and reinforcing filler (glass, quartz or ceramic oxides). Polymer phase usually comprises a relatively viscous (structurally rigid) base monomer(s) (expected to reduce PS due to its relatively large molecular volume and enhance the modulus of the cured polymer) and less viscous diluent comonomer(s) (improves handling properties and polymer conversion due to its greater flexibility and diffusion) [39]. To date, the majority of commercial dental resins involve Bis-GMA as a base monomer and TEGDMA and/or HEMA as diluent monomers. Relatively high WS, low DVC and subsequent plasticization of Bis-GMA/TEGDMA copolymers in oral milieu [40,41] requires the introduction of alternative base monomers and/or diluent monomers to overcome some of these shortcomings. The interface between these two disparate phases is complex and its properties are generally not well understood. Coupling agents (predominantly organo-silanes) are often used in composite formulations to improve the bonding between the filler and the polymer phase. From the physicochemical view-point, it is necessary to review the chemistry of the resin phase, assess the polymerization efficacy and determine the leachability of the unreacted monomers, polymerization initiators and degradation products into the oral environment. All polymeric dental composites develop internal and interfacial stresses due to PS. Typically, the mechanical performance is tied to DVC attained upon polymerization. While high DVC are desirable, the enhanced cross-linking of the polymer (normally yielding higher DVC) also results in higher PS and, thus, may lead to material failure.

Physicochemical and mechanical evaluations of Bis-GMA-, EBPADMA-, and/or UDMA-based resins formulated with various diluent and adhesive monomers revealed a complex relationship between the monomer structure, resin composition and properties in both copolymers (unfilled resins) and the ensuing ACP composites (Figure 2). These studies also indicate that fine tuning of the resins is indeed a useful tool in designing remineralizing ACP composites for different dental applications. Generally, high DVC attained in all experimental resins and their ACP composites (≥73.5%) could be attributed to the relatively high content of HEMA monomer (up to 30.4%) in the formulations. Higher reactivity of UDMA in comparison with Bis-GMA and EBPADMA [39] apparently had no bearing on DVC attained in our experimental systems. Compared to copolymer, all composites had substantially lower BFS after aqueous immersion. A reduction of approximately 50% to 60% is attributed to the uneven distribution of as-made ACP within composites. The existence of a large number of ACP-rich/polymer-depleted areas in these composites overshadowed any strengthening that may have been accomplished by changing the resin formulations. Composites that attained DVCs between 74% and 84%, expectedly, also exhibited high volumetric shrinkage upon polymerization. The measured PS values (6.2% to 7.2%) coincide with the upper threshold of PS values reported for flowable composites (3.6% to 6.0%) and/or lower threshold for adhesive resins (6.7%–13.5%) [42]. High PS values seen in our experimental systems could be, in part, attributed to a much lower ACP filler level (40 mass %) compared to up to 85% of silica-based fillers in conventional formulations. As a possible way to reduce shrinkage while maintain a satisfactory monomer conversion, inclusion of bulkier, relative low viscosity, ring-opening monomers [43,44] may need to be included in the resin phase of our composites. In ACP polymeric composites, water/polymer and water/ACP filler interactions contribute to the overall WS profiles which, in turn, by controlling the intra-composite ACP to HAP conversion, determine the kinetics of ion release and, ultimately, the remineralization potential of these materials. Slight differences in the maximum WS values seen in the formulations with varying levels of hydrophilic vs. hydrophobic comonomers did not significantly affect the ability of all composites containing 40 mass % ACP to yield supersaturated solutions highly conducive to HAP formation. In Bis-GMA vs. EBPADMA vs. UDMA series, the most favorable remineralizing conditions (the more negative values of the calculated Gibbs free energy) apparently existed in UDMA matrices containing high levels of HEMA. In these systems, a possible synergy of accessibility of ACP to the entrained water and the resulting promotion of the internal filler and the more open cross-linked network resulted in thermodynamic conditions more favorable for mineral recovery via HAP re-deposition.

When micro-leakage occurs around the restoration, bacteria, and leachable moieties may, with time, penetrate the adjacent tissues. It has been demonstrated that almost every monomeric component can be detected in the extracts of polymerized materials [45,46]. Some of these substances may elicit various biological effects, such as genetic mutations [47]. Among commonly utilized methacrylates, TEGDMA has been reported as directly mutagenic in a mammalian cell gene mutation assay, while UDMA and HEMA exhibited no mutagenicity. Therefore, for proper evaluation of any composite’s performance, DVC studies need to be accompanied with the leachability evaluations of both unfilled resins (copolymers) and the ensuing composites. The ^1^H NMR spectroscopic study of leachables from UPHM copolymers and ACP composites revealed that all constituent monomer leached out from both copolymers and composites (Figure 3) in quantities unaffected by introduction of ACP into highly cross-linked UPHM resin. Compared to the results reported in literature, detected levels of leachable UDMA from UPHM copolymers and ACP composites (0.33 to 0.51 mM) compare very well with the concentrations reported for a wide range of UDMA/TEGDMA resins (0.26 to 0.51 mM) [48]. Significantly, the levels of leachable HEMA in our experimental systems (0.02 to 0.03 mM) were one to two orders of magnitude lower than the HEMA levels detected in restorative resins (0.16 to 0.38 mM) and resin composites (up to 3.08 mM) [49,50]. This finding is especially important having in mind the fact that, in oral milieu, HEMA metabolizes into methacrylic acid known for toxicity towards human cells [51].

An in vivo mineral regeneration study (Figure 4) demonstrated a superior remineralization efficacy of ACP composites compared with the controls. In this study, aggressive demineralization protocols were used to account for cumulative effects of prolonged service in a compressed time-frame. Additionally, ACP-driven remineralization occurred throughout the depth of the lesions rather than being restricted to the near-surface region, a phenomenon characteristic for F-generated remineralization. It can, consequently be concluded that the experimental ACP composite successfully repair damaged tooth mineral repair. Its clinical efficacy is yet to be confirmed in appropriate clinical studies.

### 3.2. Airbrushed ACP Nanofibers

Airbrushing has been demonstrated as a simple, robust, safe and inexpensive method for depositing nanofibers from various polymers and incorporating ACP into scaffolds. The explored, one-step approach limits the amount of ACP well-blended into nanofibers to approximately 20 mass %. To possibly incorporate higher levels of ACP into scaffolds, systematic adjustment of the processing parameters, use of alternative low boiling point solvents and/or polymers with higher molecular mass may be prudent.

Data on the ion release from fibers indicate only limited release of Ca and P ions (Figure 5) suggesting that, despite the high aspect ratio and surface area of the nanofibers, the tested polymers could not effectively release these ions. Weight loss experiments performed in parallel to ion release studies indicate that polymers structural breakdown controlled the ion release kinetics. Further studies focusing on material chemistry and polymer degradation rates may be necessary to resolve the issue of attaining the favorable ion release from airbrushed nanofibers.

The airbrushed polymer network supported cell growth and differentiation. The hBMSCs interacted well with all polymer substrates as indicated by increased DNA content over time (Figure 7). With hBMSCs, we observed an initial decline in DNA content with 20% ACP PDLLA scaffolds. As this drop was observed early in the experiment, it could be attributed to the initial faster degradation of PDLLA polymers in conjunction with higher ion release from fibers with highest ACP load [52,53]. Others reported that a nanocomposite of PLGA and 40% ACP nanoparticles slowed the proliferation behavior of co-cultured cells with prolonged incubation (two weeks), which may be associated with advanced/progressed differentiation [54].

### 3.3. Potential for Precision Medicine

Bulk biocompatible materials in composite ACP biomedical scaffolds are known to sustain cell growth, but their bioactivity can be further enhanced by incorporating antibiotics [55], growth factors [56,57], and gene delivery systems [58] to create multi-functional properties.

At present, the release of therapeutic agents from ACP scaffolds is commonly by erosion or biodegradation, where the scaffold can be designed to provide degradation rates for predictable drug delivery. In composite tissue-engineered constructs, the properties of each component may be tuned. For example, the synthetic polymer (i.e., PLGA) component of a tissue-engineered construct can be controlled by adjusting molar ratio of acids in polymer, molecular weight of polymer, degree of crystallinity, glass transition temperature, or pH (reviewed by [59]). Moreover, the release of Ca and P ions from composite ACP scaffolds can be influenced by exposure time [60] and the percentage of ACP [23]. In PDLLA composite fibers, the highest initial and final Ca and PO_4_ cumulative ion release was recorded for the samples containing the highest Zr-ACP content.

Though there is immense potential for multi-functional scaffolds and the delivery of biomolecules via biodegradation, they have not yet gained widespread use. There is a paucity of information regarding the use of ACP hybrid scaffolds in vivo, as a tissue-engineered approach to achieve functional restoration. Challenges that are associated with developing biodegradable multi-functional scaffolds include (1) difficulty to accurately measure very small amounts of biomolecules that are released from composite scaffolds [57]; (2) overcoming rapid degradation that can occur when exogenous factors are injected in a soluble form to the injury site, by developing carriers [56]; and (3) co-mobilization of multiple substances can be challenging because of enhanced number of interactions that can take place (i.e., the presence of ACP can exert control on the release of antibiotics from the scaffold) [55]. Furthermore, the biomolecule release rate is predetermined regardless of the patients need or change in physiological circumstances.

With advancing technologies, approaches are being developed where drug release can be controlled directly, triggered by interaction with ‘smart’ material or changes in environment, or triggered by an operator with a remote device affecting the injected or implanted drug delivery system [61]. Extensive reviews have summarized technologies for remotely-triggered drug delivery systems using nano- to macro-scale materials that respond to exogenous stimuli such as light [61,62,63], ultrasound [61,64], magnetic force [61,63,64], or electrochemical processes [61]. Taken together, future research that integrates existing ACP scaffolds with on-demand controlled drug release systems holds potential to aid to enhance therapeutic effectiveness by controlling time and drug dose to match the immediate clinical need.

## 4. Materials and Methods

**Figure 9:**
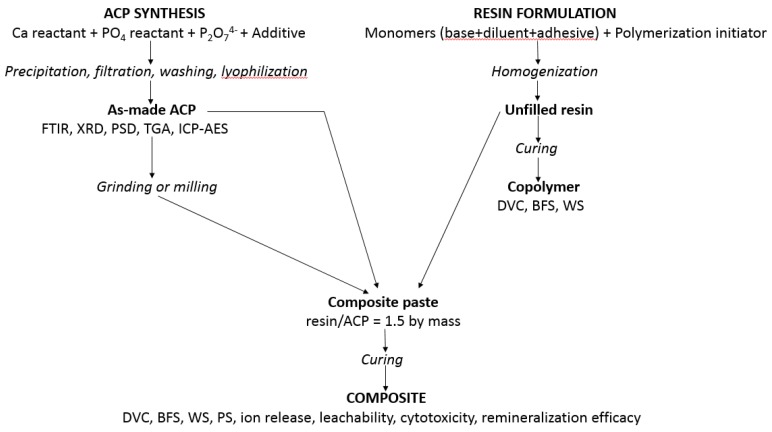
Synthetic protocols, characterization and physiochemical, mechanical, and biological evaluation of experimental resins and ACP composites.

Methods and protocols utilized to synthesize ACP, to validate/characterize fillers and resins, and to evaluate the unfilled resins (copolymers) and their ensuing ACP composites are presented in Figure 9. Detailed descriptions of these methods are provided in [22,32,36,37,38].

### 4.1. ACP Synthesis and Characterization

ACP was spontaneously precipitated from the supersaturated solutions with the initial Ca/PO_4_ molar ratio of 1.50, in the presence of 2 mol % pyrophosphate (well documented ACP stabilizer) and at pH ≥ 8.5. Additives (cations, surfactants, and/or polymers) were introduced ab initio to reduce agglomeration of ACP and yield filler with more homogeneous particle sizes. The concentrations of additives were: 10 mol % based on Ca reactant in cation series, 0.05 mass % or 0.10 mass % in surfactant series, and 0.25 mass % in polymer series. The suspensions were filtered, solids washed consecutively with ice-cold-ammoniated water (2 mass % NH_4_OH) and acetone, freeze-dried, and then lyophilized. Dry ACP was either used as-synthesized (as-ACP), or it underwent surface-modification by various silanes, grounding or milling. X-ray diffraction (XRD; DMAX 2000 diffractometer, Rigaku/USA Inc., Danvers, MA, USA) and Fourier-transform infrared spectroscopy (FTIR: Nicolet Magna-IR FTIR 550 spectrophotometer, Nicolet Instrumentation Inc., Madison, WI, USA) were employed to confirm the amorphousness of the powders. XRD patterns were recorded from 4° to 60° 2Θ with CuKα radiation (λ = 0.154 nm) at 40 kV and 40 mA. The samples were step-scanned in intervals of 0.010° 2Θ at a scanning speed of 1.000 deg/min. The FTIR spectra (4000 cm^−1^ to 400 cm^−1^) were recorded using a KBr pellet technique (0.8 mg to 1.0 mg solid/400 mg KBr). ACP powders were kept dry in a desiccator until being used in composite fabrication. For particle size distribution (PSD) experiments, ACP powders were dispersed in isopropanol and utrasonicated for 10 min at room temperature prior to the analysis (CIS-100 particle size analyzer, Ankersmid, Metropolitan Computing Corporation, East Hanover, NJ, USA). The median particle size diameter (*d_m_*) was taken as a primary indicator of the extent of particle agglomeration (the higher the *d_m_* value, the more agglomerated the ACP). Water content and the relative ratio of surface-bound vs. structurally incorporated water of ACP fillers were determined by thermogravimetric analysis (TGA; 7 Series Thermal Analysis System, Perkin Elmer, Norwalk, CT, USA). For TGA measurements, 5 to 10 mg of ACP was heated from 30 °C to 600 °C in air at the rate of 20°/min. The morphology/topology of gold-sputtered ACP powders was screened by scanning electron microscopy (JEOL 35C instrument, JEOL, Inc., Peabody, MA, USA). ACP fillers were stored under vacuum over a dessicant to avoid conversion to HAP before being used for fabrication of composites and their physicochemical, mechanical, and biological evaluations. More information on the experimental conditions employed in these protocols are provided in [65,66,67,68].

### 4.2. Resin Formulation and Evaluation

Experimental resins were formulated from the commercially available base, diluent, and/or adhesive monomers and visible light, chemical, and/or dual cure (light and chemical) polymerization initiators [22]. After combining the monomers in the required mass ratios, monomer mixtures were magnetically stirred (38 rad/s) at room temperature until achieving uniform consistency. Subsequently, the appropriate amounts of initiators were introduced to the monomer blends and the activated resin mixtures were again magnetically stirred until they became fully homogenized. The compositions of the experimental resins are enumerated in Table 1. To prevent the accidental exposure of light-cured and dual-cured activated formulations, these materials were prepared and stored in the absence of blue light. The unfilled resin (copolymer) disk specimens intended for DVC, leachability, BFS, and WS tests, and cellular assessments were prepared by filling the circular openings of flat Teflon molds (15.8–19.8 mm in diameter and 1.55–1.81 mm in thickness) with the activated resins, covering each side of the mold with a Mylar film plus a glass slide, and then clamping the assembly together with a spring clip. The light copolymer disks were photopolymerized by irradiating sequentially each face of the mold assembly for 120 s with visible light (Triad 2000, Dentsply International, York, PA, USA). The chemical cure copolymer specimens were prepared by rapidly combining equal amounts of benzoyl peroxide (BPO)- and 2,2′-dihydroxyethyl-*p*-toluidine (DHEPT)-containing resins and immediately transferring the resin mixture into Teflon molds using the same procedure as in preparation of light cure specimens without a photopolymerization step. The dual cure copolymer specimens were prepared as the chemical cure ones with the added photopolymerization step. All specimens were post-cured at 37 °C in air overnight before DVC [32,37], WS (gravimetric measurements), and BFS [69] testing.

### 4.3. Composite Formulation, Physicochemical, and Mechanical Evaluation

Composite pastes (60 mass % resin and 40 mass % ACP filler) were formulated by hand spatulation. To eliminate the air entrained during mixing, pastes were kept under a moderate vacuum (2.7 kPa) overnight before being used to fabricate composite disk specimens in a manner identical to copolymer specimen fabrication [22]. After post-curing at 37 °C in air overnight, the disks were examined by XRD to validate that the amorphous structure of the filler remained uncompromised. The composite specimens were then submitted to a battery of physicochemical, mechanical and biological tests as indicated in Figure 9.

The physicochemical characterization of the unfilled resins (copolymers) typically included DVC, BFS, and WS tests. The DVC was determined by mid-FTIR or near-IR (NIR) spectroscopy. DVC values were expressed as % reduction in the 1637 cm^−1^ absorption band for the vinyl group against that of an unchanged aromatic peak at 1538 cm^−1^ (mid-FTIR) or the percent change in the integrated peak area of the 6165 cm^−1^ methacrylate = CH_2_ absorption (NIR). Spectral data were acquired before curing and 24 h after curing by collecting 64 scans at 2 wave-number resolution. Use of an internal reference was avoided for the NIR measurements by measuring the thickness of monomer and polymer samples. The BFS values of dry (24 h in the air at 23 °C) and wet (immersion in HEPES-buffered, pH = 7.4, saline solutions at 23 °C for predetermined time intervals) disk specimens were determined using a piston-on-three-ball loading cell and a computer-controlled Universal Testing Machine (Instron 5500R, Instron Corp., Canton, MA, USA) operated by Testworks 4 software (MTS Systems, Corp., Eden Prairie, MN, USA). The BFS values were calculated according to the ASTM specification [77]. For the WS experiments, specimens were first dried over anhydrous CaSO_4_ until a constant mass was achieved (±0.1 mg) and then immersed in saline solutions (as in the BFS measurements). The mass of dry-tissue padded specimens recorded at different time intervals were used to calculate the WS of individual specimens (expressed as a % mass fraction) using a simple equation:

*WS* = [(*W_t_* − *W_o_*)/*W_o_*] × 100 (1)

where *W_t_* represents the sample mass at the time *t*, and *W_o_* is the initial value.

In addition to the DVC, WS, and BFS measurements, physicochemical tests also included PS and ion release assessments. The PS of light-cured composite specimens was measured by a computer-controlled mercury dilatometer originally developed in our group. Composite pastes were cured using a standard (60 s + 30 s) exposure and data acquisition of 60 min + 30 min. The PS of a specimen was calculated based on the known initial mass (50 to 100 mg) and its density. The latter was determined by means of the Archimedean displacement principle using an attachment to a microbalance (YDK01 Density Determination Kit; Sartorius AG, Gottingen, Germany). The release of Ca and PO_4_ ions from ACP composite disk specimens was assessed at 23 °C, in magnetically-stirred, HEPES-buffered saline solutions (25 mL saline/specimen). At predetermined time intervals, the disk specimens were taken from the immersing saline and re-immersed into “fresh” saline until next sampling. The saline solutions collected at different immersion intervals were analyzed by inductively coupled plasma-atomic emission spectroscopy (Prodigy High Emission ICP-AES, Teledyne Leeman labs, Hudson, NH, USA). The thermodynamic stability of immersion solutions was expressed as their supersaturation relative to stoichiometric HAP according to the equation:

ΔG° = −2.303 (*RT* / *n*) ln (*IAP* / *K*_sp_) (2)

where *IAP* is the ion activity product, *K*_sp_ is the HAP thermodynamic solubility product, *R* is the ideal gas constant, *T* is the absolute temperature, and *n* is the number of ions in the *IAP* (*n* = 18). The solution equilibrium program EQUIL (MicroMath Scientific Software, Salt Lake City, UT, USA) was employed in these calculations.

In vitro remineralization efficacy of composites was evaluated by quantitative microradiography on artificially-introduced enamel lesions in human teeth. The changes in mineral content of the lesions were quantified by digital image analysis of the corresponding contact microradiographs compared before and after pH cycling. The use of human teeth in remineralization studies was approved by the American Dental Association Internal Review Board and performed in compliance with the U.S. Federal Policy for the Protection of Human Subjects (institutional Federal-wide Assurance number 00023950). Specifics on experimental protocols including tooth specimen preparation, enamel demineralization, pH-cycling regimens (alternate daily exposure of specimens to demineralizing and remineralizing conditions), pre- and post-treatment analysis of contact microradiographs, and ultimate calculation of the mineral changes are provided in [36,37]. Mineral content of the lesions (ΔZ) was determined by using the commercial digital image-analysis system (Scion Image-Alpha 4.0.3.2; National Institutes of Health, Bethesda, MD, USA) interfaced with an optical microscope (Olympus BX50F; Olympus Optical Co., Ltd., Tokyo, Japan) and digital camera (RGB/YC/NTSC; Microimage Video Systems, Boyerstown, PA, USA). The relative change in mineral content, Δ(ΔZ) in %, across the depth of each lesion was calculated according to the following equation:

Δ(ΔZ) = [(ΔZ_before_ − ΔZ_after_)/ΔZ_before_]100 (3)

Positive Δ(ΔZ) values indicated mineral recovery, whereas negative Δ(ΔZ) values indicated that further mineral loss occurred as a result of the pH cycling.

An accelerated extraction approach developed in our group was employed to test for a maximum leachability of the organic moieties from copolymers and composites. The individual copolymer and composite disk specimens were fully immersed in 25–30 mL acetone (ACS quality; Fisher Scientific, Fair Lawn, NJ, USA) containing 0.01 mass % butylated hydroxy toluene, a secondary polymerization inhibitor. The extraction was performed in tightly closed containers for seven days at 23 °C with continuous magnetic stirring (32 rad/s). Specimens were then removed from the extraction solution, blotted dry, and kept for 2 h in the hood to evaporate acetone. Mass differential values of the specimens (after–before immersion) roughly indicated the amount of solvent absorbed during extraction (the exact values required correction for leachables). Each specimen was then kept under vacuum (approx. 90 kPa) for seven days at 90 °C (higher than the boiling point of acetone; i.e., 56.5 °C) to remove all of the absorbed acetone, left to cool to room temperature and its mass was recorded again. The difference between the initial dry mass and the mass after the complete acetone removal equaled total acetone-extracted leachables. The collected ^1^H NMR spectra (JEOL GSX 270 MHz Fourier transform nuclear magnetic resonance spectrometer, Peadody, MA, USA) were integrated separately for each monomer by using the polymerization inhibitor, butylated hydroxy toluene, as an internal standard. Butylated hydroxy toluene and the components of the initiator system were also analyzed to exclude their interference with the individual monomer peaks. Ultimately, the mass loss of each individual component relative to their initial amount in the formulation was calculated from their corresponding peak integration values and resulting mol % values. A more detailed description of the leachability studies is provided in [70].

### 4.4. Fabrication and Characterization of Airbrushed ACP Polymer Nanofibers

A commercially available airbrush (Master Airbrush; G222-SET; nozzle diameter 0.3 mm, pressured air 30–40 psi) was used to deposit polymer fibers from PCL (molecular mass (Mw) = 70–90,000 Da)/chloroform (4 wt % PCL)) solution, poly d,l-lactic acid (PDDLA; Mw = 115,000 Da)/acetone (8 wt % PDLLA) solution or PMMA (Mw = 500,000 Da)/acetone solution). Bulk fibers were deposited 20 cm from a target surface. For cell culture experiments, nanofibers were deposited directly onto tissue culture polystyrene disks. Zr-ACP was added (at 5 wt % or 20 wt %) to polymer solution prior to airbrushing the composite onto tissue culture polystyrene disks.

Morphological characteristics of polymer mats were obtained by scanning electron microscopy. The kinetics of ion release from the scaffolds was measured at one and four weeks of immersion of 100 mg fibers in 50 mL of continuously stirred, buffered saline solution (pH = 7.4) at 23 °C by taking aliquots and measuring Ca and P concentration by inductively-coupled plasma atomic emission spectroscopy. A total weight loss due to polymer degradation and ACP transformation upon aqueous immersion was determined gravimetrically (specimens were removed from the immersing solution and vacuum dried before weighing).

### 4.5. Cellular Studies

Cellular responses to ACP polymeric nanofibers were assessed by employing primary hBMSCs, which were maintained as described in [23].

#### 4.5.1. Double-Stranded DNA

The Quant-iT™ PicoGreen^®^ dsDNA Assay Kit (Life Technologies, Eugene, OR, USA) was utilized to estimate cell proliferation by quantifying the double-stranded DNA. The assay was performed according to the manufacturer’s instructions. Briefly, specimens were treated with lysis buffer (0.175 U/mL papain and 14.5 mM l-cysteine) to produce cellular lysate. The lysate was then transferred to a 96-well plate, mixed with of Quant-iT™ PicoGreen^®^ reagent (Life Technologies, Eugene, OR, USA) and measured using a plate reader to assess double-stranded DNA concentration (excitation 485 nm, emission 538 nm). To establish a standard curve for quantitation of the test samples, a lambda DNA standard was used to prepare serial dilutions. Cellular DNA on experimental scaffolds was quantified on cell-scaffold constructs at day 1, 16, and 50.

#### 4.5.2. Osteocalcin

Intact osteocalcin protein levels were quantified using an enzyme immunoassay kit (BT-460, Biomedical Technologies, Inc., Stoughton, MA, USA) according to the manufacturer’s instructions. Obtained data were fit to a standard curve to determine final osteocalcin concentration. The expression of osteocalcin was measured at days 16 and 50.

## Figures and Tables

**Table 1:** Compositions of the experimental resins utilizing Bis-GMA, EBPADMA, or UDMA as base monomers. A description of the acronyms is provided in Appendix A.

Constituent Monomer and/or Photoinitiator (mass %)	Bis-GMA Resins	EBPADMA Resins	UDMA Resins
Bis-GMA	36.5–68.4	-	-
EBPADMA	-	16.8–49.6	-
UDMA	-	-	34.8–92.4
TEGDMA	0–49.5	0–67.2	0–50.2
HEMA	0–29.2	0–30.4	0–29.5
HmDMA	0–46.6	0–49.3	0–47.3
EHMA	24.0–34.0	-	-
PEG-U	-	-	0–29.1
MEP	-	0–5.0	0–2.9
Zr-DMA	0–1.0	-	-
BPO	-	-	0–2.0
Camphorquinone	0–0.2	0.2	0.2
CGI	0–3.0	-	-
4265 DAROCUR	0–0.8	0–0.8	0–0.8
DHEPT	-	-	0–1.0
4EDMAB	0–0.8	0–0.8	0–0.8
1850 IRGACURE	0–1.0	0–1.0	0–1.0
369 IRGACURE	0–1.5	0–1.5	0–1.5

## Appendix A. List of Acronymns

1850 Irgacure	bis(2,6-dimethoxybenzoyl)-2,4,4-trimethylpentyl phosphine oxide & 1-hydroxycyclo-hexyl phenyl ketone
369 Irgacure	2-benzyl-2-(dimethylamino)-1-(4-(4-morphollinyl)phenyl)-1-butanone
4265 Darocur	diphenyl(2,4,6-trimethylbenzoyl) phosphine oxide & 2-hydroxy-2-methyl-1-phenyl-1-propanone
ACP	amorphous calcium phosphate
Ag-ACP	silver-modified ACP
Al-ACP	alumina-modified ACP
ANOVA	analysis of variance
BFS	biaxial flexural strength
Bis-GMA	2,2-bis[p-(2-hydroxy-3-methacryloxypropoxy)phenyl]propane
BPO	benzoyl peroxide
Ca	calcium
CaP	calcium phosphate
CGI	bis(2,6-dimethoxybenzoyl)-2,4,4-trimethylpentyl phosphine oxide & 2-hydroxy-2-methyl-1-phenyl-1-propanone
DHEPT	2,2′-dihydroxyethyl-*p*-toluidine
DVC	degree of vinyl conversion
EBPADMA	ethoxylated bisphenol A dimethacrylate
EHMA	ethyl-α-hydroxymethyl acrylate
4EDMAB	ethyl-4-*N*,*N*-dimethylamino benzoate
Fe(II)-ACP	iron (II)-modified ACP
Fe(III)-ACP	iron (III)-modified ACP
FTIR	Fourier transform infrared spectroscopy
ΔG°	Gibbs free energy
HAP	hydroxyapatite
hBMSC HEMA	human bone marrow stromal cell 2-hydroxyethyl methacrylate
HmDMA	hexamethylene dimethacrylate
IAP	ion activity product
Ksp	thermodynamic solubility product
MEP	methacryloyloxyethyl phthalateMEP
Mw	molecular mass
*n*	number of specimens (or repetitive experiments)
NIR	near infrared spectroscopy
NMR	nuclear magnetic resonance
PCL	polycaprolactone
PDLLA	poly-d,l-lactic acid
PEG-U	poly(ethylene glycol) extended urethane dimethacrylate
pH	−log (molar concentration of hydrogen ions)
PMMA	poly(methyl methacrylate)
PO_4_	phosphate
PS	polymerization shrinkage
R	ideal gas constant
SD	standard deviation
Si-ACP	silica-modified ACP
*T*	absolute temperature
TEGDMA	triethylene glycol dimethacrylate
UDMA	urethane dimethacrylate
UPHM	UDMA/PEG-U/HEMA/MEP resin
WS	water sorption
Zn-ACP	zinc-modified ACP
Zr-ACP	zirconia-modified ACP
